# Individual and Contextual Factors Associated with Adolescents’ Self-Perceived Need for Treatment

**DOI:** 10.3390/ijerph21040395

**Published:** 2024-03-24

**Authors:** Roanny Torres Lopes, Érick Tássio Barbosa Neves, Laio da Costa Dutra, Ramon Targino Firmino, Larissa Chaves Morais de Lima, Saul Martins Paiva, Fernanda Morais Ferreira, Ana Flávia Granville-Garcia

**Affiliations:** 1 Graduate Program in Dentistry, Department of Dentistry, State University of Paraíba, Campina Grande 58429-500, Paraíba, Brazil; roannytorres@gmail.com; 2 Department of Dentistry, State University of Paraíba, Araruna 58233-000, Paraíba, Brazil; ericktassiobarbosaneves@gmail.com; 3 São Francisco College, Cajazeiras 58900-000, Paraíba, Brazil; laiodutra@gmail.com; 4 Academic Unit of Biological Sciences, Federal University of Campina Grande, Patos 58708-110, Paraíba, Brazil; ramontargino@gmail.com; 5 Faculty of Medical Sciences of Campina Grande, UNIFACISA University Center, Campina Grande 58408-326, Paraíba, Brazil; larissachaves@outlook.com; 6 Department of Child and Adolescent Oral Health, Federal University of Minas Gerais, Belo Horizonte 31270-901, Minas Gerais, Brazil; smpaiva@uol.com.br (S.M.P.); femoraisfe@gmail.com (F.M.F.)

**Keywords:** oral health, schools, family relations, adolescent, dental health services

## Abstract

The present study aimed to investigate associations between the self-perceived dental treatment need and clinical factors, familial characteristics, and school context in adolescents. A cross-sectional study was conducted with a representative sample of 746 students aged 15 to 19 years in a medium-sized city in Brazil. Data collection involved the use of a sociodemographic questionnaire, an oral health questionnaire, and the Family Adaptability and Cohesion Scales (FACES III) instrument. Clinical examinations were performed by two trained and calibrated examiners (Kappa > 0.80) using the Nyvad criteria. A robust logistic regression analysis for complex samples was performed using a multilevel approach (α = 5%). The individual factors associated with the self-perceived treatment need were dental pain (OR = 1.08; 95% CI: 1.01–1.16), the loss of the first molars (OR = 1.09; 95% CI: 1.03–1.15), and disengaged family cohesion (OR = 1.15; 95% CI: 1.01–1.31). In terms of context, attending a public school was associated with the self-perceived treatment need (OR = 1.17; 95% CI: 1.02–1.33). Thus, the individual factors of toothache, tooth loss, and a disengaged family, as well as the school context, exerted an influence on the self-perceived treatment need.

## 1. Introduction

Self-perceived dental treatment need is the subjective assessment of one’s own oral health status based on what one recognizes as healthy. This parameter has been scarcely explored in the literature but is an indicator of oral health, as the perception of treatment need exerts an influence on self-care and dental service-seeking behavior [1]. The study of factors associated with the self-perceived treatment need is important in adolescence as the supervision of guardians lessens in this phase, and oral health self-care may be neglected. Thus, adolescents may be vulnerable to oral health problems [2,3]. 

Studies on factors associated with the self-perceived dental treatment need have been directed at clinical factors, such as dental caries in the anterior and posterior regions, tooth loss, pain, advanced periodontal disease, esthetic issues, dissatisfaction with one’s teeth, dental appointments for the purpose of treatment, and oral health-related quality of life [1,4,5,6]. The perception of caries in the anterior region is important as this location is more easily visible, and attention can be given early to avoid disease progression. The extant literature does not provide details on the number of carious lesions on anterior teeth. However, such information could contribute to the understanding of disease severity and commitment to oral health care. Moreover, the loss of a permanent first molar is a relevant issue, as this is the first permanent tooth to erupt in the oral cavity [2] and often does not receive adequate care. 

Besides clinical factors, it is important to investigate the influence of non-clinical factors, such as the family and school contexts, on the self-perceived dental treatment need. The family contributes to the formation of health-related habits in childhood that extend into adolescence and adulthood [7]. Thus, studies have focused on the influence of family type on oral health outcomes and indicators, demonstrating that family cohesion can exert an influence on the oral health status and treatment need among students [5,8]. Less cohesive families have worse oral health outcomes due to the greater difficulty in sharing healthy habits among their members [7]. Thus, a more in-depth understanding of the influence of the family context on the self-perceived treatment need in adolescents is warranted. 

The school setting also plays an important role in establishing health behaviors and can impact the quality of life of adolescents [9]. To date, no studies have evaluated the influence of the school context on the self-perceived dental treatment need in adolescents. Such knowledge is important to guide strategic activities that strengthen adolescents’ awareness of their oral health. The conceptual hypothesis of this study is that family cohesion (individual factor) and the type of school (context) influence the self-perceived dental treatment need in adolescents.

Previous studies examining self-perception regarding the need for dental treatment primarily focused on assessing variables related to socioeconomic and clinical aspects [1,5]. This study aimed to expand this scope by integrating non-clinical factors into the analysis. Specifically, we investigated the influence of family relationships (referred to as family cohesion), a factor previously established as affecting adolescents’ health-related decisions, as well as the impact of the school type, which serves as a predictor of adolescent behavior, life choices, and health habits. The inclusion of these additional variables in this study provides a unique perspective and underscores the importance of considering diverse contextual influences on adolescents’ health evaluations [10,11]. Consequently, these findings offer valuable insights into understanding the multifaceted nature of adolescents’ health perceptions, highlighting the significant role played by environmental contexts in influencing the self-perception of oral health in this group. Therefore, the present study aimed to investigate clinical and non-clinical factors that influence the self-perception of oral health in adolescents. 

## 2. Materials and Methods

The Human Research Ethics Committee of Universidade Estadual da Paraíba approved this analytical cross-sectional study (approval number: 55953516.2.1001.5187), and it adhered to the ethical guidelines established in the Declaration of Helsinki. Data were gathered from October 2016 to July 2017 from adolescents aged 15 to 19 years residing in Campina Grande, located in the state of Paraíba, Brazil. Thirty-two schools, comprising 16 public and 16 private institutions, were randomly selected to represent the distribution of adolescents across the city’s six administrative districts. Within these schools, adolescents were chosen through simple random sampling. Participation required parental or guardian consent and the assent of the adolescents, who each provided their agreement by signing an informed consent form and an assent form, respectively. 

The sample size of the study was determined via an analytical approach involving the comparison of two independent proportions using the G* Power software program, version 3.1 (Franz Faul, Universität Kiel, Kiel, Germany), with a significance level of 95% and a power of 80%. In the pilot study, the proportions that reported a need for dental treatment were 84% in public school and 72.3% in private schools. Consequently, a minimum sample size of 390 adolescents was calculated. Cluster sampling was performed in two stages. The schools were first randomly selected, and then, the participants were chosen using a simple random sampling procedure. Since this type of sampling tends to produce a more homogeneous sample, a correction factor was employed to increase heterogeneity. Correction factors typically range from 1.2 to 2.0, and for this study, a factor of 1.6 was applied, resulting in a sample size of 624 adolescents [12,13]. An additional 20% was added to account for potential dropouts [14], bringing the final target sample size to 780 adolescents.

The study excluded adolescents with physical, sensory, or behavioral limitations that would avoid or exert a negative impact on the reading and completion of the forms or interfere with the clinical variables. Additionally, adolescents undergoing orthodontic treatment were also excluded from the study. 

### 2.1. Data Collection Instruments

Sociodemographic data (the adolescent’s sex, skin color, and type of school; the mother’s schooling; and the monthly family income) were collected using a questionnaire completed by the adolescent’s parents/guardians [15]. Income was categorized based on the median, considering the Brazilian monthly minimum wage at the time of data collection (USD 240).

On a second occasion, adolescents were taken to a designated room in their schools at prearranged times to complete printed questionnaires regarding their oral health and family cohesion/adaptability. The oral health questionnaire was structured around queries from the latest Brazilian National Oral Health Survey^15^. Dental pain was assessed using the question, “Have you experienced toothache in the past 6 months?” with the response options of “No” or “Yes”. The self-perceived need for dental treatment was evaluated with the question, “Do you believe you currently require dental treatment?” offering the response choices of “Yes”, “No”, and “I don’t know”.

The Family Adaptability and Cohesion Scales (FACES III) instrument was used to assess the adolescent’s family relationship. This tool was developed by researchers from the University of Minnesota and defines family cohesion as the capacity of a family to remain united in the face of day-to-day changes. The FACES III instrument comprises 20 items with five possible answers (almost never = 1, rarely = 2, sometimes = 3, often = 4, and almost always = 5), which were explained by the examiner before the questionnaire was administered [16,17]. The included questions are easy to understand for teenagers aged 12 and over.

The odd-numbered items refer to cohesion, and the even ones inquire about adaptability. According to the response pattern, families are classified into one of the following categories: disengaged (low cohesion and high independence among the members), separated (moderate-to-low cohesion and a certain degree of independence), connected (moderate-to-high cohesion and moderate dependence), or enmeshed (high cohesion and dependence) [16,17]. 

### 2.2. Clinical Examination 

After the adolescents completed all the questionnaires, they were transferred to a reserved room designated by the school’s administration for a clinical examination. Before the oral examination commenced, all the adolescents received a toothbrush with fluoridated toothpaste and performed supervised brushing. Afterward, they were seated in front of the examiner, who used a sterile gauze to dry the teeth and a mouth mirror, a millimeter probe (OMS-621, Trinity^®^, Campo Mourão, PA, Brazil), and a Petzl Zoom headlamp (Petzl America, Clearfield, UT, USA) to perform the clinical exam. A trained assistant recorded the findings on an appropriate chart. For those with active white spots, acidulated phosphate fluoride (APF) with a concentration of 1.23% (12,300 ppm) was applied. Additionally, all participants received a toothbrush and counseling on oral hygiene.

The Nyvad criteria were used for the diagnosis of dental caries on the anterior teeth. These diagnostic criteria allow for the identification of caries at various stages, from initial to most advanced. This index involves visual and tactile examination and includes the following categories: (0) sound tooth, (1) carious lesion with intact surface, (2) active lesion with interrupted surface, (3) active lesion with cavitation, (4) inactive lesion with intact surface, (5) inactive lesion with interrupted surface, (6) inactive lesion with cavitation, (7) restoration in good condition, (8) restoration with active lesion, and (9) restoration with inactive lesion. In this study, only active caries on anterior teeth (central incisors, lateral incisors, and canines), corresponding to codes 1, 2, and 3, were considered [18]. Tooth loss due to caries was clinically assessed, and adolescents were asked to provide details about any missing teeth to rule out other causes.

### 2.3. Training and Calibration

The training and calibration for using the Nyvad index used the appropriate methodology for epidemiological studies on dental caries [19]. For this, a theoretical step was initially performed, which consisted of the presentation and study of the diagnostic criteria and clinical routine. Then, a practical step was performed, consisting of the assessment of clinical images of carious teeth. Two researchers were trained and calibrated by a dentist with experience with the use of the Nyvad criteria. The results were discussed with the experienced dentist, who served as the “gold standard”. Afterward, 50 adolescents were clinically examined for the determination of inter-examiner agreement (Kappa: 0.89–0.90). After 7 days, the procedure was repeated for the determination of intra-examiner agreement (Kappa: 0.88–0.90).

### 2.4. Pilot Study

Before commencing the main study, 50 adolescents were conveniently selected from a public school (n = 25) and a private school (n = 25) to assess the suitability of the proposed methods for achieving the study’s objectives. The findings indicated no necessity to modify the methods. These adolescents were not included in the final study sample.

### 2.5. Statistical Analysis

Data were analyzed using descriptive and inferential statistics. Descriptive analysis was conducted to characterize the sample using SPSS Statistics, with a confidence level of 95% (SPSS for Windows, version 22.0, SPSS Inc., Chicago, IL, USA).

Robust logistic regression for complex samples was employed to identify variables associated with the outcome (self-perceived treatment need). The outcome was associated with both individual-level (first-level) and school-level (second-level) variables. Odds ratios (ORs) were computed to indicate the likelihood of an event occurring within the studied group. A null model was utilized to assess the behavior of the variables in the model and as a data-adjustment parameter. Individual variables with a *p*-value < 0.20 in the unadjusted model (sex, monthly family income, tooth pain, loss of the first molar, family cohesion, and number of anterior teeth with active caries) were included in Model 2. Subsequently, the contextual-level variable (type of school) was integrated with the individual variables into Model 3. Variables with a *p*-value < 0.05 in the final model were deemed to be associated with the outcome. Deviance values (−2 log likelihood) were used to assess the goodness of fit of the models.

## 3. Results

The final sample was composed of 746 adolescents. The approximately 4% loss from the target sample was due to the absence of adolescents on the days of the examination after three attempts. The adolescents were predominantly female (59.5%), with non-white skin color (71.7%) and a family income less than or equal to the monthly minimum wage (51.0%); see Table 1.

The prevalence of active caries on anterior teeth with a discontinuous surface, active caries with an intact surface, and active caries with cavitation, respectively, was 6.6%, 51.1%, and 39%.

In the unadjusted analysis, dental pain, tooth loss, the number of teeth with carious lesions, family cohesion, and type of school were associated with the self-perceived treatment need (Table 2).

In the logistic regression analysis, the following variables were associated with the self-perceived dental treatment need: tooth pain (OR = 1.08; 95% CI: 1.01–1.16), loss of molars (OR = 1.09; 95% CI: 1.03–1.15), disengaged type of family cohesion (OR = 1.15; 95% CI: 1.01–1.31), and attending a public school (OR = 1.17; 95% CI: 1.02–1.33) as the contextual variable (Table 3).

## 4. Discussion

The present study investigated whether clinical factors, family characteristics, and the school context are associated with the self-perceived need for dental treatment. The conceptual hypothesis of the present study was confirmed. Adolescents who attended public schools and belonged to families with the disengaged type of family cohesion were more likely to report a self-perceived treatment need compared to those who attended private schools or belonged to enmeshed families. Moreover, clinical factors, such as dental pain and the loss of first molars, influenced the self-perceived dental treatment need in the studied adolescents. These results highlight the intersectoral role of prevention measures and oral health promotion in this group, including actions that involve the school setting, family, and healthcare providers in the planning and execution of oral health care [8]. 

The prevalence of the self-perceived need for treatment was 88.6%. Previous studies on populations in the same age range showed lower prevalences (62.6–67.6%) [1,4]. These studies mainly addressed clinical characteristics, such as dental pain, tooth decay, periodontal disease, malocclusion, and changes in tooth color, also demonstrating that the greater the impact of these clinical factors on the quality of life regarding oral health, the greater the perception of the need of dental treatment [4]. A possible explanation for the difference between our study and the cited investigations may be related to oral health conditions and the availability of oral health services. Possibly, adolescents included in the present study had worse oral health conditions and less access to dental care, leading to more accumulated dental treatment needs. This scenario may have contributed to the greater self-perceived need for treatment. 

In the adjusted analysis, active caries on anterior teeth were not associated with the self-perceived treatment need. On the other hand, previous studies analyzing clinical factors found an association between tooth decay in the anterior and posterior teeth and the self-perceived treatment need in adolescents in the same age group [1,5]. The prevalence of active caries on anterior teeth was 57.7%, and the mean number of caries on anterior teeth was low 1.62 (2.04). The initial appearance of these lesions (white spots) and the low number of cavitated lesions may have contributed to the non-recognition of the presence of caries. Previous studies have pointed out that the perception of incipient lesions may be hampered, especially in the initial stages, when only white spots are present [20,21]. Likewise, cavities in the early stages may not cause sensitivity or pain and remain unperceived, thus delaying treatment [22]. Another study that evaluated the presence of caries found a high prevalence thereof (92.8%) among adolescents in the same age group [8]. In this case, the greater number of carious lesions and lesions in advanced stages could contribute to the greater perception of dental treatment needs. This underscores the importance of investing in educational campaigns that stimulate periodic visits to a dentist to combat caries in the initial stages.

Symptomatic and functional parameters, such as toothache and the loss of the first permanent molars, also exerted an influence on the self-perceived treatment need, a result similar to that found in other studies [1,5]. This finding demonstrates that adolescents are more affected when the condition is more evident, affecting both esthetics and function. Dental pain is the most common consequence of tooth decay and the reason for seeking a dentist [23], in addition to compromising the quality of life of adolescents and impairing the performance of daily activities [23]. It has already been demonstrated in the literature that adolescents have more difficulty seeking dental treatment and tend to delay appointments and prioritize curative measures over preventive measures [24]. Difficulty chewing, caused by pain or the loss of first molars, can generate discomfort, suggesting a preferentially symptomatic self-perception pattern [23,25,26]. It has also been shown that dental pain and the need for treatment are associated with greater dissatisfaction with oral health [6].

Adolescents from disengaged families (greater independence among the members) had a greater likelihood of self-perceived dental treatment needs compared to those with a high degree of family cohesion. As demonstrated in previous studies, family relations influence the oral health of adolescents and health-related attitudes. Families with a high degree of cohesion were observed to have a higher prevalence of cavities, which also contributes to visits to the dentist [8,26]. In another study, a low-to-moderate level of family cohesion was associated with a greater presence of carious lesions [8]. This finding is in line with our study, demonstrating that adolescents still lack family support in building a balanced level of health. It is possible that by not having greater closeness among family members, oral diseases follow their course with no preventive/interceptive intervention, increasing the severity of the oral condition and, consequently, the self-perceived treatment need. There is less supervision by parents and more incentive for greater independence in the final years of adolescence. Moreover, there is encouragement to search for one’s first job, and adolescents are responsible for making decisions and taking care of their own health [27]. This result is important and suggests the need to strengthen public policies that guide adolescents to seek adequate dental services. However, it is also important to strengthen family cooperation in the propagation of health care measures, as neglect in care can delay the resolution of unsatisfactory oral conditions [7]. 

In terms of the school context, adolescents attending public schools perceived a greater need to undergo dental treatment. Although socioeconomic factors, such as family income, were not associated with the outcome in this study, the type of school is a proxy for family income in Brazil. Attending a public school suggests a low income, and not all such schools have ideal conditions or disease-prevention programs [10]. A previous study demonstrated that adolescents at public schools have less knowledge about prevention, worse hygiene habits, and a poorer oral health status compared to those at private schools [11]. Another previous study demonstrated that adolescents who studied in public schools and had a higher school retention rate had a higher risk of having untreated cavities [28]. Furthermore, the prevalence of visits to the dentist is lower in schools that offer incentives for prevention [29]. These are important points, as the school context plays an important role in preventing adverse health conditions in the population, and school programs can influence health patterns and acquired behaviors [30]. Therefore, the analysis of the entire context in which the adolescent is inserted is important to understand its repercussions for oral health.

Adolescent health is a complex issue, as it involves the interaction of social, emotional, psychological, and physical factors, as well as the influence of different contexts, such as access to health services, which can reduce adolescents’ vulnerability to health problems [24]. Contrary to expectations, the condition of the anterior teeth, considered an aesthetic region, was not relevant for the adolescents to perceive the need for treatment. Self-perception has been associated with conditions resulting from the progression of oral diseases. There is possibly a lack of effective communication with adolescents regarding prevention and the signs that indicate that they need to seek treatment.

The present study has limitations that should be mentioned. First is the inability to assess the effect of temporality due to the study’s cross-sectional design. Another limitation is that the results may have been influenced by response bias, as we relied on self-reported data for a number of variables. On the other hand, the strengths of the paper are also worth mentioning. Cross-sectional studies, such as the current one, are important for investigating factors associated with the outcome studied and identifying priorities for the development of public health policies. Moreover, measures of methodological rigor were adopted in the present investigation to confer internal reliability to the results, such as the conduction of a pilot study and training of the examiners, as well as sample size calculation and selection of the sample by clusters, to ensure the external validity of the data.

## 5. Conclusions

We conclude that adolescents with a history of tooth pain, those who had lost the first permanent molar, those belonging to disengaged families, and those from public schools were significantly more likely to report self-perceived dental treatment needs. Oral health preventive strategies, as well as dental care, should be prioritized for adolescents with this profile. Future studies are needed to deepen the understanding of the influence of the school context and family environment on the self-perceived need for treatment.

## Figures and Tables

**Table 1:** Characterization of adolescents 15 to 19 years of age.

Quantitative Variable	Mean (Standard Deviation)	
**Number of anterior teeth with active caries**	1.62 (2.04)	
**Categorical variables**	**Frequency**	
	N	%
**Sex**		
Female	444	59.5
Male	302	40.5
**Mother’s schooling ^a^**		
≥8 years of study	443	59.7
<8 years of study	299	40.3
**Monthly family income ^b^**		
>USD 240	261	49.0
≤USD 240	272	51.0
**Self-declared skin color**		
White	211	28.3
Non-white	535	71.7
**Tooth pain ^c^**		
No	456	61.9
Yes	281	38.1
**Loss of first molar**		
No	640	85.8
Yes	106	14.2
**Family cohesion**		
Disengaged	344	46.1
Separated	266	35.7
Connected	121	16.2
Enmeshed	15	2.0
**Self-perceived treatment need ^d^**		
No	77	11.4
Yes	597	88.6
**Contextual variable**		
**Type of school**		
Private	249	33.4
Public	4 97	66.6

^a^ Missing data for four responses. ^b^ Missing data for 213 responses. ^c^ Missing data for nine responses. ^d^ Missing data for two responses.

**Table 2:** Unadjusted analysis of self-perceived treatment need in adolescents.

Self-Perceived Treatment Need			Unadjusted OR **	
Categorical Variables	Yes n (%)	No n (%)	95% CI	*p*-Value
**Sex**				
Female	363 (90.3)	39 (9.7)	1.00	-
Male	234 (86.0)	38 (14.0)	1.05 (0.99–1.11)	0.09
**Mother’s schooling**				
≥8 years of study	345 (58.0)	47 (61.8)	1.00	-
<8 years of study	250 (42.0)	29 (38.2)	1.01 (0.96–1.07)	0.51
**Monthly family income**				
>USD 240	202 (87.1)	30 (12.9)	1.00	-
≤USD 240	235 (91.4)	22 (8.6)	1.05 (0.98–1.11)	0.11
**Self-declared skin color**				
White	165 (88.7)	21 (11.3)	1.00	-
Non-white	432 (88.5)	56 (11.5)	0.99 (0.93–1.06)	0.94
**Tooth pain**				
No	341 (85.3)	59 (14.8)	1.00	-
Yes	249 (93.3)	18 (6.7)	1.10 (1.03–1.17)	<0.001
**Loss of first molar**				
No	496 (86.7)	1 (1.0)	1.00	-
Yes	101 (99.0)	76 (13.1)	1.11 (1.06–1.17)	<0.001
**Family cohesion**				
Disengaged	282 (89.5)	33 (10.5)	1.10 (1.05–1.15)	<0.001
Separated	208 (86.3)	33 (13.7)	0.99 (0.90–1.08)	0.83
Connected	94 (89.5)	11 (10.5)	0.97 (0.90–1.04)	0.47
Enmeshed	13 (100.0)	0 (0.0)	1.00	-
**Contextual variable**				
**Type of school**				
Private	176 (83.0)	36 (17.0)	1.00	-
Public	421 (91.1)	41 (8.9)	1.09 (1.02–1.17)	<0.001
**Quantitative variable**	**Mean (SD)**	**Mean (SD)**		
**Number of anterior teeth with active caries**	1.04 (1.86)	0.51 (1.02)	1.26 (1.07–1.49)	<0.001

** Independent controlling variables of the unadjusted analysis.

**Table 3:** Adjusted multilevel analysis of individual and contextual variables associated with self-perceived need for dental care in adolescents.

	Model 2 OR (95% CI)	*p*-Value	Model 3 OR (95% CI)	*p*-Value
Intercept	0.79 (0.70–0.88)		0.73 (0.62–0.85)	
Individual variables				
**Sex**				
Female	-	-	-	-
Male	1.02 (0.94–1.10)	0.630	1.02 (0.94–1.11)	0.517
**Monthly family income**				
>USD 240	-	-	-	-
≤USD 240	1.11 (1.02–1.20)	0.012	1.02 (0.95–1.10)	0.470
**Tooth pain**				
No	-	-	-	-
Yes	1.07 (1.00–1.16)	0.049	1.08 (1.00–1.16)	0.039 *
**Loss of first molar**				
No	-	-	-	-
Yes	1.12 (1.06–1.19)	< 0.001	1.09 (1.03–1.15)	0.003 *
**Family cohesion**				
Disengaged	1.11 (0.99–1.25)	0.061	1.15 (1.01–1.31)	0.032 *
Separated	0.97 (0.86–1.09)	0.652	0.99 (0.87–1.12	0.894
Connected	0.98 (0.89–1.07)	0.672	0.99 (0.90–1.09)	0.934
Enmeshed	-	-	-	-
**Number of anterior teeth with active caries**	1.01 (0.98–1.04)	0.452	1.00 (0.97–1.03)	0.619
**Contextual variable**				
**Type of school**				
Private	-	-	-	-
Public	-	-	1.17 (1.02–1.33)	0.018 *
**Deviance (−2 log likelihood)**	48,528.58		48,456.88	48,456.88

Model 1 (“null”) presents the unconditional model; intercept: 0.88 (0.84–0.91) and deviance: 65,112.42; Model 2 presents individual covariates; Model 3 presents individual and contextual-level covariates. * Significance level: *p* < 0.05.

## Data Availability

The data presented in this study are available on request from the corresponding author.
